# High herbivore pressure favors constitutive over induced defense

**DOI:** 10.1002/ece3.2208

**Published:** 2016-07-29

**Authors:** Ryan J. Bixenmann, Phyllis D. Coley, Alexander Weinhold, Thomas A. Kursar

**Affiliations:** ^1^ Department of BiologyUniversity of Utah257S 1400ESalt Lake CityUtah84112; ^2^ Smithsonian Tropical Research InstituteBox 0843‐03092BalboaRepublic of Panama; ^3^ German Centre for Integrative Biodiversity Research (iDiv), Halle‐Jena‐LeipzigDeutscher Platz 5eLeipzig04103Germany

**Keywords:** Defense chemistry, induced defenses, phenolics, plant–herbivore interactions, saponins, secondary metabolites, tropical forest

## Abstract

Theoretical and empirical studies show that, when past or current herbivory is a reliable cue of future attack and defenses are costly, defenses can be induced only when needed and thereby permit investment in other functions such as growth or reproduction. Theory also states that, in environments where herbivory is constantly high, constitutive defenses should be favored. Here, we present data to support the second aspect of the induced resistance hypothesis. We examined herbivore‐induced responses for four species of *Inga* (Fabaceae), a common canopy tree in Neotropical forests. We quantified chemical defenses of expanding leaves, including phenolic, saponin and toxic amino acids, in experimental field treatments with and without caterpillars. Because young leaves lack fiber and are higher in protein than mature leaves, they typically lose >25% of their leaf area during the few weeks of expansion. We predicted that the high rates of attack would select for investment in constitutive defenses over induction. Our data show that chemical defenses were quite unresponsive to herbivory. We demonstrated that expanding leaves showed no or only small increases in investment in secondary metabolites, and no qualitative changes in the phenolic compound profile in response to herbivory. The proteinogenic amino acid tyrosine, which can be toxic at high concentrations, showed the greatest levels of induction. *Synthesis*: These results provide some of the first support for theoretical predictions that the evolution of induced vs. constitutive defenses depends on the risk of herbivory. In habitats with constant and high potential losses to herbivores, such as tropical rainforests, high investments in constitutive defenses are favored over induction.

## Introduction

**Figure 1:**
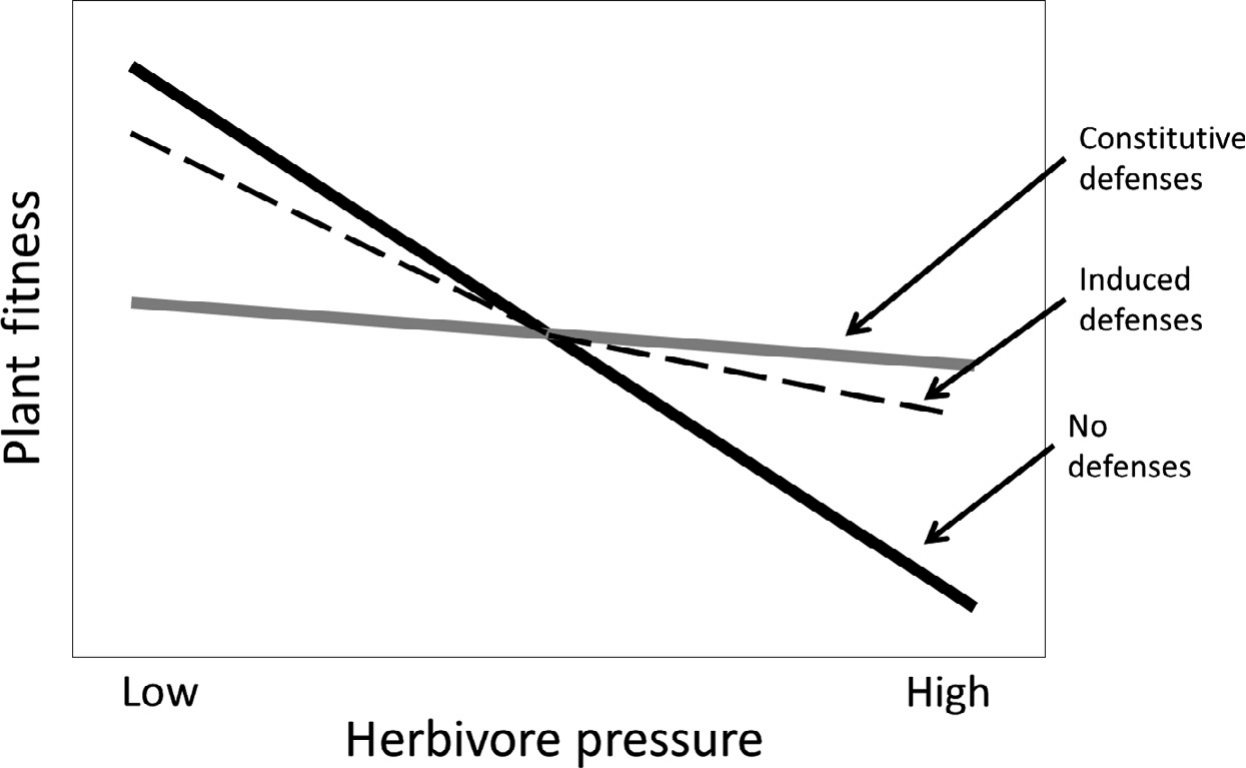
Fitness under a range of herbivory environments for two defense regimes. Adapted from Karban et al. (1999). Species that evolved with low or intermittent herbivore pressure will have higher fitness if they rely on induced defenses whereas species that have evolved with high or constant herbivore pressure will have higher fitness using constitutive defenses.

Plants have evolved a wide diversity of antiherbivore defenses (Johnson 2011; Mithöfer and Boland 2012) and face an inherent dilemma in how they invest in those defenses (Herms and Mattson 1992). High investment in defense may result in reduced loss to herbivores, but also reduced resources for growth or reproduction. Empirical evidence has demonstrated that the cost of investing in defense can be quantified in reduced growth, lower photosynthetic production, and reduced plant fitness (Redman et al. 2001; Moore et al. 2003; Preisser et al. 2007). The fitness consequences of investing in constitutive defense are context dependent and are addressed by several hypotheses, including optimal defense, resource availability, and carbon/nutrient balance (McKey 1974, 1979; Rhoades 1979; Bryant et al. 1983; Coley et al. 1985). These hypotheses predict that plants maximize their fitness by balancing allocation to constitutive defense versus other functions based on available resources, herbivore pressure, tissue value, and cost of defense (Stamp 2003). Under conditions of low herbivore pressure, plant fitness would be maximized by investing little in constitutive defenses. In contrast, under conditions of high herbivore pressure, the fitness benefits of constitutive defenses could outweigh the fitness costs (Fig. 1).

In contrast to constitutive defense, induced defense is considered a cost‐saving strategy in which defenses are expressed only in response to herbivory. Consequently, induced defenses are predicted to evolve when herbivore pressure is variable and when current or past herbivory is a reliable predictor of future attack (Karban and Adler 1996; Karban and Baldwin 1997; Karban et al. 1997, 1999). Thus, under conditions of variable herbivore pressure, a strategy of induced defenses would lead to greater fitness than a strategy of constitutive defenses (Fig. 1). However, under high and constant herbivore pressure, plants with induced defenses would have a lower fitness than plants with constitutive defenses due to herbivore damage that occurs during the delay in the induction of a defense and the costs associated with signaling cascades (Fig. 1). Thus, the induced resistance hypothesis predicts that, under high, constant herbivore pressure, continuously expressed constitutive defenses would be more adaptive than induced defenses (Zangerl and Rutledge 1996; Karban and Baldwin 1997; Karban 2011).

Here, we examine induction of defenses in a genus of Neotropical rainforest trees (*Inga*) and experimentally manipulate herbivory on young leaves using lepidopteran larvae in a field setting. While young leaves are expanding, they are tender and high in protein, two traits that make them a target for herbivores. Once leaves reach full size, they rapidly toughen, and rates of herbivory drop to almost zero (Kursar and Coley 2003). Thus, young leaves receive 70% of the leaf's lifetime herbivore damage despite being vulnerable for only a few weeks (Kursar and Coley 2003). Under such high herbivore pressure, even a delay of 1 day to induce defenses could result in substantial damage (Aide and Londoño 1989). Hence, induction may not be rapid enough in order to provide effective defenses (Karban and Adler 1996). Taken together, these observations suggest that, for expanding leaves in these high herbivore conditions, plants will use constitutive defenses rather than induced defenses. As a further test of the extent of plasticity of defense expression in expanding leaves, we also studied the effect of light, a factor that strongly influences the chemistry of mature leaves (Bryant et al. 1983).

We chose *Inga* for this study of induced defenses because its chemical and nonchemical defenses have been extensively characterized (Kursar et al. 2009). *Inga* has over 300 species and is one of the most species‐rich and locally abundant tree genera in Neotropical forests (Kursar et al. 2009). The defensive traits for the common species of *Inga* at our study site, Barro Colorado Island, Panama (BCI), are diverse and include chemical, developmental, phenological, and biotic defenses (Koptur 1984, 1985; Coley et al. 2005; Kersch and Fonseca 2005; Lokvam and Kursar 2005; Brenes‐Arguedas et al. 2006; Lokvam et al. 2006; Brenes‐Arguedas et al. 2008; Coley & Kursar 2014). Moreover, in this study, we have complemented gravimetric analyses of chemical defenses with metabolomic analyses. The chemical defenses of *Inga* include triterpene saponins, polymers of flavan‐3‐ols with diverse substitutions, polygalloylated compounds, and the protein amino acid, tyrosine. These secondary metabolites have shown toxicity to Lepidoptera in experimental feeding trials (see Figures S1–S4 in Supporting information). The recent divergence and high diversity of defensive traits among closely related *Inga* species in a single tropical forest (Kursar et al. 2009; Endara et al. 2015) make it an ideal system for investigating the effects of environmental factors on defense expression.

## Materials and Methods

### Study site

Field research was conducted on BCI from May to November (rainy season) 2007 and 2008. BCI is located in the Panama Canal (9°N 80°W) and is administrated by the Smithsonian Tropical Research Institute. The island is a tropical moist lowland forest that experiences a 4‐month dry season (January to April, Leigh 1999). For *Inga*, most young leaves are produced during the rainy season (P. D. Coley & T. A. Kursar, unpubl. data). This is also the season that herbivores and their predators are most abundant in both gaps and understories (Richards and Windsor 2007).

### Field experiment

To assess whether herbivores could induce defenses in expanding leaves, we randomly assigned *Inga* saplings along the trails of BCI to either an herbivore damage treatment (plus‐caterpillar) or an herbivore‐free treatment in which herbivores were removed and they received no herbivore damage (minus‐caterpillar). Minus‐caterpillar plants were visited daily to maintain their leaves free of herbivores. Because mechanical wounding alone does not mimic herbivore damage (Arimura et al. 2005), we used a common herbivore that feeds on all the *Inga* species on BCI, a leaf roller (Gelechiidae, species not identified, Kursar et al. 2006). Second and third instar gelechiids were collected from nonfocal *Inga* plants and moved to plus‐caterpillar saplings of four species of *Inga*.

All four *Inga* species are shade‐tolerant trees that range from 20–35 m tall. *Inga marginata, I. pezizifera* and *I. umbellifera* are widely distributed throughout Central and South America in a diversity of lowland forest microhabitats (Pennington 1997). *Inga multijuga* is distributed throughout lowland tropical forests in Central America near water and swamps (Pennington 1997). This species was identified as *I. multijuga* in the BCI flora (Croat 1978), but recent, unpublished revisions suggest it may be *Inga thibaudiana* (R. Perez, T.D. Pennington, C. Kidner, J. Nichols, pers. comm.). We chose these particular four species of *Inga* because they are locally abundant and differ in their metabolic profiles of secondary metabolites.

*Inga* have extra‐floral nectaries that produce sugar during leaf expansion and attract predacious ants that protect the leaves. Previous research showed that ant presence increased nectar production, but that herbivore presence had no effect (Bixenmann et al. 2011). To test whether ants affect expression of chemical defenses by plants, herbivore treatment was crossed with an ant treatment. Individual plants either received normal ant visitation to leaves (plus‐ants) or ant access to leaves was restricted by the addition of a sticky barrier (Tanglefoot) on the branch (minus‐ants). In addition, plants were evenly sampled in both gaps and in the understory to determine the effect of light level and other microclimatic differences on chemical defenses. These three factors (herbivore, canopy and ant treatment) were fully crossed for each species and an individual plant only experienced one level of each treatment.

The treatments were applied to saplings between 1 and 4 m tall. Plants were incorporated into the experiment before the leaves reached 15% of their average adult leaf area. Plants with preexisting damage on the young leaves were not used, and study plants were never used twice. In addition, minus‐caterpillar plants that had accumulated damage during the experiment were not used for analysis. Damage to the minus‐caterpillar treatment was always <0.4% of the focal leaf area and was 0.05% on average. Second and third instar caterpillars were placed on young leaves (<15% full size) at the start of the experiment and allowed to fed for the next 5–9 days, until the leaf was harvested. Plus‐caterpillar plants that received <5% damage on their focal leaves were not used. Treatments were maintained until leaves reached 80% of their adult size (the size when the majority of herbivory has occurred and defense chemicals are high, Kursar and Coley 2003) or until over half of their leaf tissue was damaged. At these terminal points, leaves were collected for laboratory analysis. Leaves were clipped from the tree and placed in a paper envelope in the field and within 5 h were vacuum‐dried and stored at −20°C until chemical analysis. The final sample sizes for each species after leaves were removed for insufficient or excess damage were as follows: *I. multijuga* (*n* = 27), *I. pezizifera* (*n* = 22)*, I. marginata* (*n* = 28)*,* and *I. umbellifera* (*n* = 28).

### Chemical analyses

Two classes of chemicals (phenolics and saponins) and one amino acid (tyrosine) were extracted, separated, and quantified gravimetrically. These chemicals were selected based on their toxicity to herbivores in previous feeding trials using extracts of *Inga* species (Figures S1–S4; Potter and Kimmerer 1989; Agrell et al. 2003; Coley et al. 2005). *I. marginata, I. multijuga,* and *I. pezizifera* all contain polyphenols composed of gallocatechin/galloepicatechin gallate. In addition, *I. marginata* contains oleanolic acid‐based saponins and *I. pezizifera* contains echinocystic acid‐based saponins. *I. umbellifera* contains polyphenols based on cinnamoylated pyranosides of catechin/epicatechin. In *I. umbellifera,* tyrosine is over‐expressed and accumulates to 10% DW. In feeding trials with a generalist herbivore, tyrosine levels of 3.8% DW were toxic, reducing caterpillar growth by 50% (Figure S4; Lokvam et al. 2006).

For *I. marginata*,* I. multijuga*, and *I. pezizifera*, 70–80 mg of sample was homogenized using grinding beads in a 1‐mL Nunc Cryo Tube™ and a Wig‐l‐bug^®^ grinding mill (REFLEX Analytical Corporation, Ridgewood, NJ) at 46 Hz for a total of 3 min. The grinding beads were removed and 1 mL of 80% ethanol was added and mixed. Samples were then centrifuged for 10 min at 9055 RCF and 5°C. The supernatant was retained, and the extraction was repeated a total of five times with 80% ethanol. The same process was repeated twice with 70% acetone, and all collected supernatants were combined. Pellet and extract were dried under nitrogen, then under a vacuum (0.8 torr) at ambient temperature and weighed.

To remove lipids, 3 mL of 60% methanol (MeOH) and 3 mL of hexane were added to each extract. Vials were shaken and allowed to settle. Once two distinct layers formed, the lipid‐containing hexane layer was removed and placed in a preweighed vial. Next, 3 mL of hexane was added, and the separation was repeated for a total of five times. Both the polar organic fraction and lipids were dried under nitrogen and then under a vacuum (0.8 torr) at ambient temperature and weighed.

The polar organic fraction was separated on a liquid chromatography column packed with Bakerbond reverse phase octadecylsilane (ODS). Columns were prepared in 10‐mL syringes plugged with glass wool and filled with 1.9 g of ODS. Water–methanol solutions were used to serially elute the columns as follows: 30 mL of 5% MeOH (low molecular weight, polar molecules), 10 mL of 5% MeOH (blank), 20 mL of 60% MeOH (phenolics), 10 mL of 60% MeOH (blank), 20 mL of 100% MeOH (saponins), and 10 mL of 70% acetone (blank). Each fraction was collected separately into a labeled preweighed vial, dried under nitrogen, and then under a vacuum (0.8 torr).

Fractions were quantified gravimetrically, and the class of compounds in each fraction was verified using a Hitachi LaChrom Elite HPLC (Hitachi High Technologies America, Dallas, TX) with an Omnisphere C18 250 × 2.0 mm column (Varian‐Chrompack, Middelburg, The Netherlands), a diode array detector and evaporative light‐scattering detector (Sedere S.A., Alfortville, France). The blank fractions were used to verify separation among fractions; these contained <5% of the total mass and were not included in the analyses.

Further chemical characterization of the phenolics of *I. marginata*,* I. multijuga*, and *I. pezizifera* was accomplished using UPLC‐MS. The dried residues of the polar fraction were dissolved in 1 mL 60% MeOH and sonicated for 20 min to yield a concentration of approximately 10 mg/mL and transferred to a HPLC vial. The samples were diluted 1:2 with MeOH and 2 mL were injected in ACQUITY I‐Class UPLC (Waters Corporation, Milford, MA) equipped with a Waters BEH C18‐column (50 mm × 2.1 mm × 1.7 *μ*m). The column temperature was kept constant at 40°C, and the solvents were MS grade (Fisher Scientific, Waltham, MA). Solvent A was water + 0.1% formic acid (MS grade, Sigma Aldrich, St. Louis, MO), solvent B was acetonitrile + 0.1% formic acid, and the following gradient was applied: 95% A for 1 min, 10 min 85% A, 20 min 50% A, 25 min 5% A, 30 min 95% A, and 35 min 95% A. The flow rate was 0.3 mL/min. Mass spectra were acquired in positive ESI resolution mode on a XevoG2 (Waters Corporation, Milford, MA) equipped with a lock spray source using the following parameters: m/z range 50–1200 Da, capillary voltage 2.9 kV, sampling cone 40 V, extraction cone 4 V, source temperature 120°C, desolvation gas temperature 400°C, desolvation gas flow 900 L/h, and a collision energy of 6 eV as set by the manufacturer for optimizing ion optics without fragmentation. The acquisition rate was 1 Hz, the instrument was calibrated to a sodium formate standard, and leucine enkephalin was used as reference on the lock spray. Five replicates per treatment were analyzed.

The acquired data files were converted to NetCDF format using the MassLynx software and the msconvert plug‐in by applying the “SortByScanTime” filter. NetCDF files were processed by the XCMS R‐package (Smith et al. 2006) to detect features (consisting of a certain retention time and m/z range), that are different between *Inga* species or treatments. The “centWave” algorithm with the following parameters was used for signal processing: ppm = 10, peakwidth c (5, 20), and snthresh = 10. Peak matching parameters were as follows: method = “density”, bw = 10, mzwid = 0.05, and minfrac = 0.6. Peak alignment parameters were as follows: method = “density”, bw = 5, mzwid = 0.05, and minfrac = 0.1. These steps converted the raw data into a list of over one thousand peaks or “features”, each with a specific retention time and m/z value along with a total ion count for each feature in every extract. Further data processing is described under “Statistics”.

### Tyrosine and phenolics in *Inga umbellifera*

Due to the high quantity of tyrosine in *I. umbellifera*, and tyrosine's low solubility, a special extraction protocol was developed. Twenty‐five milligrams of dried leaf sample were homogenized as above and extracted in 2 mL of acidified 10% MeOH (90% water adjusted to pH = 3 with acetic acid/10% MeOH; v/v) for 20 min at 80°C. Samples were centrifuged at 13,250 × *g* and ambient temperature for 5 min. The resulting supernatant was retained, and the extraction was repeated. The combined supernatants were separated on preweighed Agilent SampliQ C18 solid‐phase extraction columns (500 mg ODS). The supernatant was added to the prepared column and washed with an additional 2 mL 10% MeOH (pH 3). The 10% MeOH wash contained tyrosine and was dried under vacuum (0.8 torr) and redissolved in 20 mL of 10% MeOH. Samples were then separated on a Hitachi LaChrom Elite with an Omnisphere C18 250 × 2.0 mm column isocratically using 10% MeOH/90% HOH with 0.1% formic acid. Tyrosine was detected at 275 nm and quantified based on peak area and external calibration curves. The SampliQ columns were dried and reweighed. As *I. umbellifera* does not contain saponins (Coley et al. 2005), the difference in final minus initial weights was considered to be the mass of the phenolic fraction trapped on the column.

### Statistical analyses

Individual plants were treated as units of replication. When a plant had samples from multiple leaves, the values for the multiple collections were averaged for an individual plant. Only values from leaves that were in the targeted size range (60–90% of adult size) were used. The LC‐MS data are summarized in Appendix S1. The proportions of the three chemical defenses (phenolics, saponins, and tyrosine) within a young leaf were arcsine‐transformed prior to analysis to meet the assumptions of ANOVA. Analyses of variance were run for each defense compound (Table 1) using herbivore presence, ant presence, canopy, and young‐leaf size as explanatory variables with an alpha level of 0.05. *Inga* species was used as a blocking variable. Nonsignificant parameters were removed from each model using the “step” function in R (R Development Core Team 2009). All main effects and interaction terms were included in the original model. “Step” then creates all possible models with one term removed from the model and compares the Akaike information criteria (AIC) values. Step then passes the new model with the lowest AIC (i.e., best fit) to another iteration of model selection until no better fit can be found. For tyrosine (in *I. umbellifera*), plant species was not used because it was found in only one species.

**Table 1:** ANOVA table for the percent of leaf dry weight of phenolics, saponins, and tyrosine. The table includes main effects and interactions. Plant species was used as a blocking variable to remove variation due to different natural history traits

Factor	df	Sum sq.	Mean sq.	*F*‐value	*P*‐value
Phenolics					
Herbivore presence	1	0.02185	0.021845	9.8848	<0.01**
Ant presence	1	0.002	0.002003	0.9064	0.344
Canopy	1	0.0035	0.003496	1.582	0.212
Young‐leaf size	1	0.03167	0.031673	14.3316	<0.001***
Expansion rate	1	0.03337	0.033367	15.0981	<0.001***
*Inga* species (Block)	2	0.62103	0.310515	140.5052	<0.001***
Herbivore presence × Ant presence	1	0.00313	0.003128	1.4154	0.238
Ant presence × Canopy	1	0.00716	0.007164	3.2414	0.075
Herbivore presence × Young‐leaf size	1	0.00273	0.002726	1.2334	0.27
Herbivore presence × Expansion rate	1	0.01048	0.010477	4.7406	<0.05*
Ant presence × Expansion rate	1	0.00309	0.003089	1.398	0.24
Young‐leaf size × Expansion rate	1	0.01718	0.017176	7.7721	<0.01**
Young‐leaf size × *Inga* species	2	0.02055	0.010273	4.6485	<0.05*
Ant presence × Canopy × Expansion rate	2	0.01134	0.005668	2.5646	0.083
Herbivore presence × Young‐leaf size × Expansion rate	1	0.01813	0.018132	8.2044	<0.01**
Canopy × Young‐leaf size × Expansion rate	2	0.00607	0.003036	1.3736	0.259
Saponins					
Herbivore presence	1	0.000009	0.000009	0.011	0.917
Ant presence	1	0.00205	0.002051	2.4817	0.123
Canopy	1	0.06724	0.067236	81.3444	<0.001***
Young‐leaf size	1	0.01566	0.01566	18.9462	<0.001***
*Inga* species (Block)	1	0.27048	0.270483	327.2416	<0.001***
Herbivore presence × Ant presence	1	0.00372	0.003723	4.5043	<0.05*
Young‐leaf size × *Inga* species	1	0.00462	0.004616	5.5841	<0.05*
Tyrosine					
Herbivore presence	1	0.03724	0.037235	14.4512	<0.01**
Ant presence	1	0.00011	0.000109	0.0423	0.839
Canopy	1	0.08868	0.088677	34.416	<0.001***
Young‐leaf size	1	0.02836	0.028362	11.0076	<0.01**
Herbivore presence × Ant presence	1	0.01415	0.014147	5.4907	<0.05*
Herbivore × Young‐leaf size	1	0.00715	0.007147	2.7738	0.111

*P < 0.05, **P < 0.01, ***P < 0.001.

For the mass spectrometry data, as some features with retention times >22 min showed evidence of contamination, all features >22 min were removed from the peak list. Also removed were features that were missing in three or more samples per treatment group and features representing contamination from polyethylene glycol. This reduced the dataset to 550 features. As datasets with features having zero ion counts cannot be analyzed, a value of 1 was added to every feature. The data were normalized by dividing all of the ion counts for each sample by the ion count for the 75 percentile (upper quartile normalization), a method to avoid biased detection of up‐ or downregulated signals in gene expression data (Fundel et al. 2008). The normalized dataset was submitted to the MetaboAnalyst web server for ANOVA, volcano plots, PCA, and hierarchical clustering analyses (Xia et al. 2012). This R‐based online platform was used to rescale the data using Pareto scaling, a method for avoiding bias from very highly expressed features (e.g., lowers the relative contribution of large values or large changes; van den Berg et al. 2006). Features that were significantly different between treatments after the volcano plot analysis (Figure S5) were inspected manually in the chromatograms and checked for their intensity.

## Results

### Herbivory

The realized herbivory rates in our experiments averaged 0.05% leaf area lost, with a range of 0–0.4% for the minus‐caterpillar treatment and averaged 27%, with a range of 5.1–66% for the plus‐caterpillar treatment.

### Phenolics

**Figure 2:**
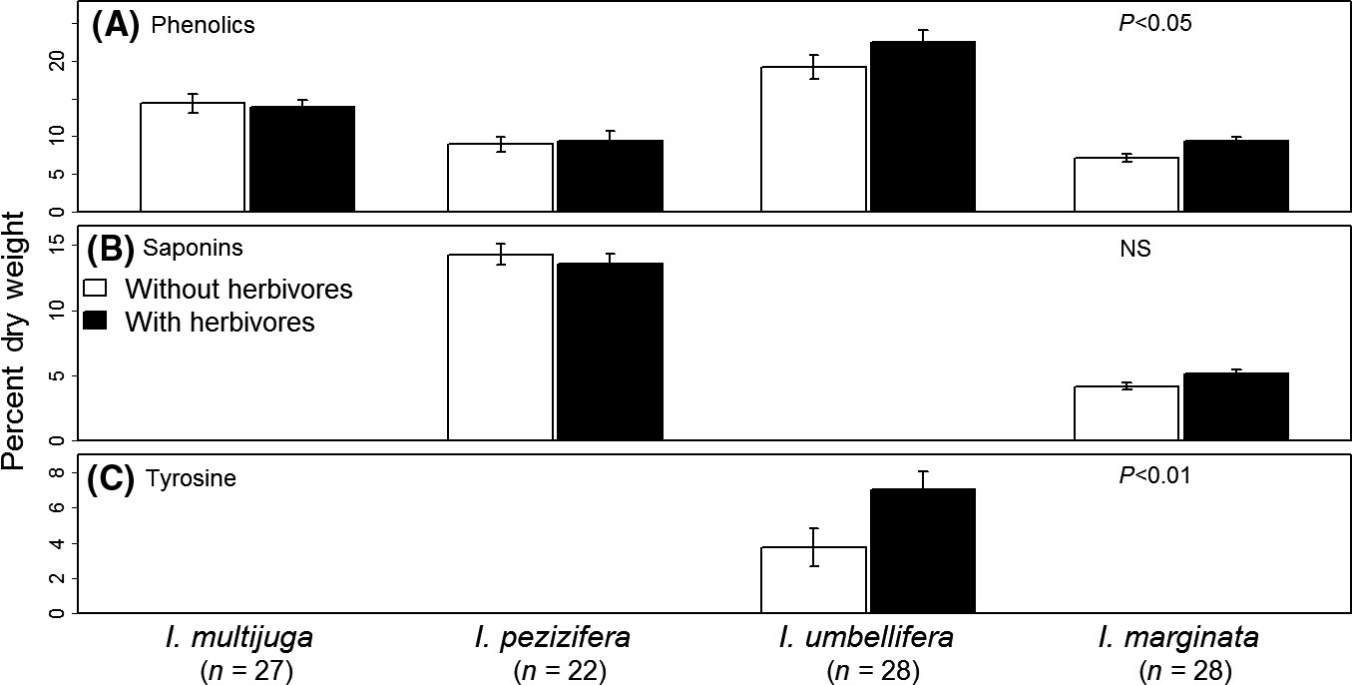
Herbivore presence induced the phenolic fraction and tyrosine within the young leaves of four species of *Inga*. The *y*‐axis is the percent dry weight (mg of fraction/mg dry weight of leaf) for three principal chemical defenses in *Inga*: (A) phenolics, (B) saponins, and (C) tyrosine. Open bars are individuals from which herbivores were excluded, and solid bars are individuals to which herbivores were artificially added. The sample size is included in parentheses below each species along the *x*‐axis.

Phenolic content ranged from 4% to 41% of leaf dry weight (DW) across the four *Inga* species. Overall, there was a small, but, significant effect of herbivores on phenolic mass (*F*_1, 84_ = 9.88, *P* < 0.01, Fig. 2A). Furthermore, there was a significant interaction between herbivore presence and the rate of leaf expansion (*F*_1, 84_ = 4.74, *P* < 0.05, Table 1). Herbivory had no effect for two slow‐expanding species, *I. multijuga* and *I. pezizifera*, but caused an increase in phenolics of 21% and 33% in the two fast‐expanding species, *I. marginata* and *I. umbellifera*. This was equivalent to an absolute increase of 2.4% DW and 4.1% DW, respectively.

Ant presence and light environment (canopy cover) did not induce phenolic compounds in young‐leaf tissue (Table 1, Figures S6A and S7A). In three cases, younger leaves had a higher phenolic concentration than older leaves. Specifically, in *I. multijuga*,* I. umbellifera*, and *I. marginata*, phenolic content decreased by 25%, 35%, and 20%, respectively, from leaves in size class 3 (50–74% of adult size) to size class 4 (75–100% of adult size, *F*_1, 84_ = 14.33, *P* < 0.001, Figure S8A). Phenolic contents did not differ by leaf age for *I. pezizifera* (Figure S8A).

### Saponins

The saponins in *I. pezizifera* and *I. marginata* were 14% and 5% of DW, respectively. These were not induced by herbivores (Fig. 2B) or ants (Table 1, Figure S6B). However, saponin content was 9% (*I. pezizifera*) and 6% (*I. marginata*) higher when plants were found in gaps (*F*_1, 42_ = 81.34, *P* > 0.001, Figure S7B) and, as with phenolic compounds, saponin content decreased by 20% from size class 3 young leaves to size class 4 in *I. marginata* (*F*_1, 42_ = 18.94, *P* > 0.001, Figure S8B), but not in *I. pezizifera*. *I. multijuga* and *I. umbellifera* did not contain saponins (Fig. 2B).

### Tyrosine

Only *I. umbellifera* had tyrosine in toxic amounts (4% DW). For tyrosine, the responses to light and herbivores and the changes with leaf age were substantial. Tyrosine was induced by 97% when herbivores were present (*F*_1, 20_ = 14.45, *P* < 0.01, Fig. 2C). That is equivalent to an increase of 3.6% DW (from 3.7% DW to 7.3% DW). Tyrosine content increased by 148% in gaps (*F*_1, 20_ = 34.42, *P* < 0.001, Figure S7C) and decreased by 147% from size class 3 young leaves to size class 4 (*F*_1, 20_ = 11.01, *P* < 0.01, Figure S8C).

### Metabolomics

**Figure 3:**
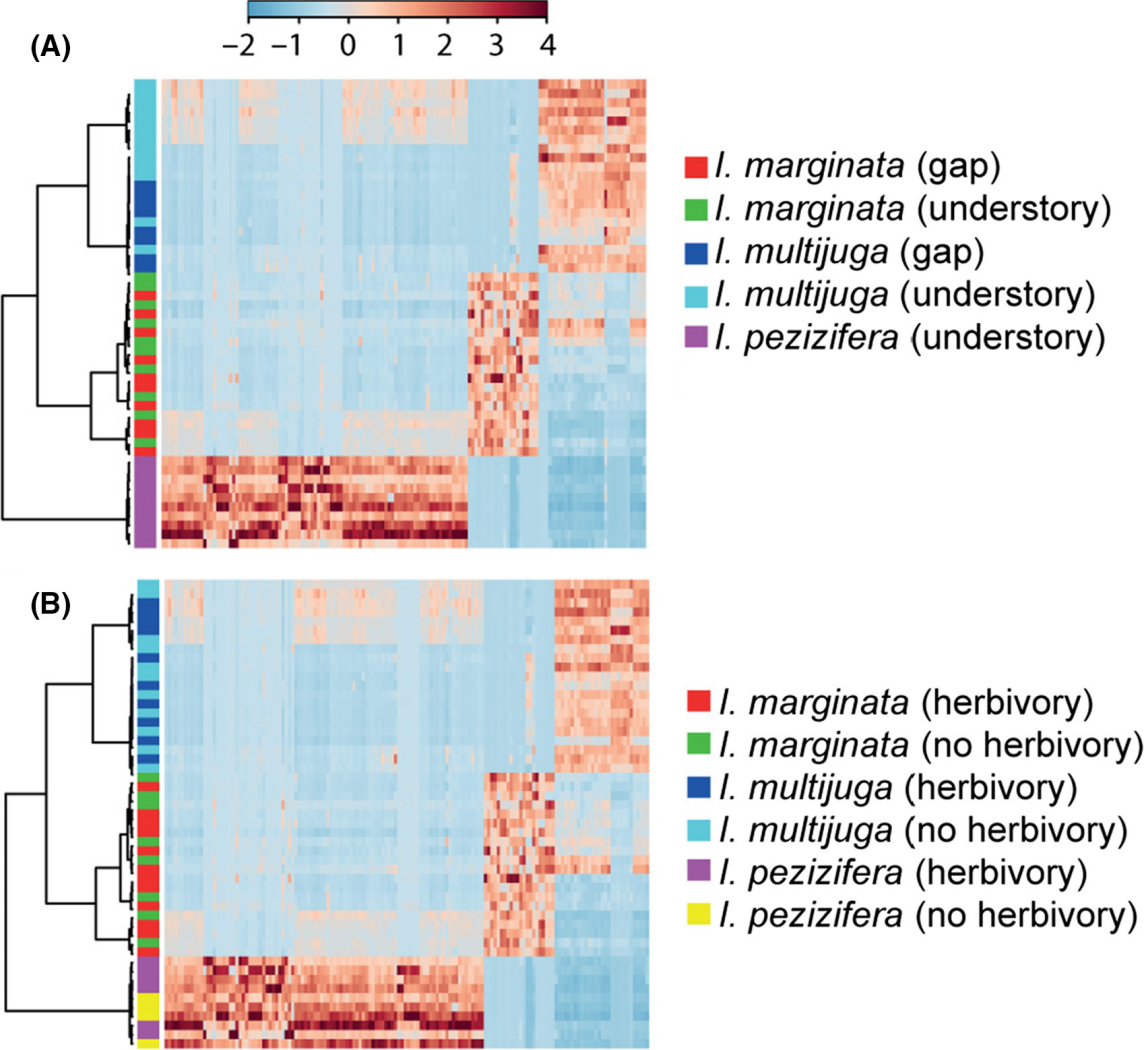
Heatmap comparison of the influence of light and herbivory on the metabolic profile of different *Inga* species (each row is a different plant). The top significant (ANOVA) features were selected to build the trees (each column is a feature). Ward linkage and Spearman distance algorithms were used. (A) Comparison of *Inga marginata, I. multijuga,* and *I. pezizifera in* gap and understory environments. (B) Comparison of *Inga marginata, I. multijuga,* and *I. pezizifera* with and without herbivory. No differences are observable between treatments, but species are clearly distinguished by their metabolite profile.

For three species, we analyzed the qualitative composition of the fraction containing phenolic compounds using metabolomics (UPLC‐MS). The metabolome did not change in response to herbivore presence or light (Figs. 3, S9 and S10). That is, each of these *Inga* species had a unique combination of phenolic compounds or saponins (Figure S9A), but, overall, the presence and absence of those compounds did not change among herbivore treatments (Figs. 3B, S9B, and 10C, D).

Because some effects may be missed in a multivariate analysis, we specifically examined the effects of herbivory on individual features. We found a negligible number of features that changed more than twofold (*P* ≤ 0.05; Figure S5). Inspection of these features in the chromatograms showed that most were false positives (Table S1), features extracted by the peak‐picking algorithm in XCMS, but representing low abundance peaks or background noise. In summary, none of the known defensive compounds of *Inga* were identified in this analysis of individual features, suggesting that herbivory on expanding leaves does not induce specific phenolic compounds or saponins.

## Discussion

### Induction of secondary metabolites by herbivory

The amount of damage, as well as the timing and the identity of the damage agent, can significantly impact induced responses. For example, many species are induced by damage well below 20% of total leaf area (Karban and Baldwin 1997) and some species show induced responses to damage to 5% of their leaf area (Moore et al. 2003). Hence, an alternative interpretation of the observed, limited induction is that expanding leaves in the minus‐caterpillar treatment had damage sufficient to induce a full response (Heil and Baldwin 2002). But, this would suggest that nearly all leaf flushes in tropical rainforests receive damage that is sufficient for induction. Our plants were inspected daily for caterpillars and damage, and, although some minus‐caterpillar plants had damage, it was very low (0.05%). This was almost three orders of magnitude lower than the plus‐caterpillar treatment (27%) and, moreover, for a leaf of 100 cm^2^, damage of 0.05% would be about 2 mm in diameter. Hence, the most parsimonious explanation is that plants in the minus‐caterpillar treatment did not receive a signal sufficient to cause induction and that the observed responses of the plus‐caterpillar plants represent the response to herbivory.

Regarding the identity of the damage agent, the most critical issue is that using herbivores, as in our study, induces a stronger response than mechanical damage (e.g., scissors). The other key factor that causes variability in induced defenses is the length of time allowed for plants to induce. Whole‐plant induction of defenses typically starts within 24 h and can reach maximum levels by 4 days (Ohnmeiss et al. 1997; Marti et al. 2013). In our experiment, caterpillars were feeding on plants was for 5–9 days as the leaf grew from <15% to ~80% of full size (see “Materials and Methods”). Thus, our study had a time period that was sufficiently long to permit the accumulation of induced defenses.

Despite substantial amounts of damage, herbivory had no effect on the profile of phenolic compounds (i.e., qualitative change) for any of the three species analyzed (Figs. 3B, S9B and S10C, D). Herbivory also had no effect on the mass of phenolic compounds (i.e., quantitative change) for two species, but caused an induced increase of 27% in the other two species (Fig. 2A). This is at the low end of the levels of induction by herbivores in other woody species. The majority of reports describe a 10–60% induction of phenolics in mature leaves of temperate species (Wagner and Evans 1985; Hartley and Firn 1989; Erwin et al. 2001; Baraza et al. 2004; Moreira et al. 2009, 2014). Although two studies reported no detectible induction of phenolics (Wold and Marquis 1997; Massei et al. 2000) despite significant herbivore damage, others have reported a 2.2‐ to 7.5‐fold (i.e., 120–650%) induction of phenolics from herbivore or artificial damage (Schultz and Baldwin 1982; Baldwin and Schultz 1983; Dutsadee and Nunta 2008). However, the average absolute increase in % DW for these same reports was 2.7% DW, which is similar to the highest increase we saw in *Inga* (3.2% DW).

There is less information for induction in tropical species. There was no detectible induction in woody plants in South African savannahs (Bryant et al. 1991) nor for 17 rainforest trees (Cárdenas et al. 2015). However, in a study of dry forest species with a design similar to ours, treatments were applied during leaf expansion and leaves were collected at the end of leaf expansion (Boege 2004). Two of three species showed an increase in the relative abundance of total phenolics when not protected from herbivores. These were 16% (about 5% more of total DW) and 29% (about 3% more of DW) for *Croton pseudoniveus* and *Bursera instabilis*, respectively. These increases are comparable to our gravimetric results for phenolics for the two species that induced 21% and 33% (“Results” and Fig. 2A). For condensed tannins in *B. instabilis*, the content more than doubled, 1.1% and 3.2% of leaf DW, for protected vs. unprotected plants, respectively (Boege 2004). For the legume *Bauhinia brevipes* in cerrado, the tannin content of recently matured leaves did not differ between leaves that were protected vs. exposed to herbivores (Cornelissen and Fernandes 2001). Interestingly, leaves with simulated damage had twice the tannin content found in the other two treatments. Phenolics in the mature leaves of *Shorea leprosula* (a wet forest species) were induced by 55% (5% DW), but only in forest gaps and not in the understory (Massey et al. 2005). Our results are the first to report on induced defenses in expanding leaves from a tropical forest. Comparison with the above studies of mature leaves suggests relatively less induction in expanding leaves than in mature leaves following herbivory.

In *I. marginata* and *I. pezizifera* (the two saponin‐containing species), saponins were not induced by herbivores. Agrell et al. (2003) reported for alfalfa (*Medicago sativa*: Fabaceae) that herbivory induced a 53% increase in antifungal activity and reduced caterpillar feeding. In contrast, our data indicate that *Inga* saponins are constitutively expressed in the expanding leaves. Aside from the present study and Agrell et al. (2003), we are not aware of other studies of saponin responses to herbivory. In contrast to phenolics and saponins, tyrosine did show a substantial increase in response to herbivory (97%).

### Physiological constraints on induction

Our hypothesis that an environment with consistently high damage would lead to the evolution of primarily constitutive defenses is supported by these results. Additionally, leaf expansion is rapid enough that induction may occur too late in development to be beneficial (Karban and Adler 1996). But, we also consider other mechanisms that may make induction less likely. For example, the increase of tyrosine throughout development and its postexpansion decline (Lokvam et al. 2006; Bixenmann et al. 2013) suggest that, as is well established in other plants, tyrosine can be redirected into other primary or secondary metabolites. Thus, induced production of tyrosine would not be a “lost” investment as the plant could recycle the tyrosine. In contrast, saponins, phenolics, and particularly condensed tannins may not be easy to catabolize and recycle. If this is the case, accumulating an excess of these metabolites in young leaves may not be adaptive as they would persist once the leaf was full size and protected by toughness (Coley 1983; Coley et al. 1985; Lucas et al. 2000). Therefore, in contrast to tyrosine, physiological constraints on catabolism may select against induction of phenolics and saponins.

Another constraint on induction may be the very high baseline investment in constitutive secondary metabolites, a total of about 50% of DW when cell wall‐bound phenolics are included (Lokvam and Kursar 2005; Wiggins et al. 2016). For highly metabolically active tissue, this may be the upper limit of investment. Hence, further increases could exceed the capacity to store these metabolites in a manner that avoids autotoxicity (Agrawal and Karban 1999). Thus, although constitutive and induced defenses are not considered to be opposing traits in some agricultural and artificial settings (Leimu and Koricheva 2006; Kempel et al. 2011), that is, both may occur together, our data support the hypothesis that there is a trade‐off or a negative correlation between investment in constitutive and induced defenses under natural conditions and with “wild” species (Zangerl and Rutledge 1996; Koricheva et al. 2004; Zhang et al. 2008; Kempel et al. 2011; Moreira et al. 2014; Rasmann et al. 2015).

Other factors have been reported to affect induction of defenses, but we think they are not likely in our system. For example, reproductive demands of adult trees can suppress induction, but this would not be a factor in the juvenile plants in our study. Induction in young leaves due to current damage to mature leaves is also unlikely as herbivory on mature leaves is extremely low (Kursar and Coley 2003). In temperate systems, it has been suggested that early season herbivory might cause induction in later season growth (Karban and Adler 1996). As an individual sapling in a tropical forest will produce leaf flushes that are separated by several months for gap plants to 1 year for shaded plants, such an effect is possible.

### Changes in secondary metabolites in response to light

The carbon–nutrient balance hypothesis predicts that plants in high light should increase carbon‐based defenses over nitrogen‐based defenses (Bryant et al. 1983). For the two species for which we had plants from treefall light gaps and the understory, metabolomics analyses showed no qualitative change in the phenolic profile of young leaves in response to light (Figs. 3A, S9C and S10A, B). In terms of quantitative changes, we found modest increases in total DW investment, similar to a study of mature leaves of *Inga oerstediana* that reported an increase in phenolic content of 23% (Nichols‐Orians 1991). Another study of expanding leaves of *Inga paraensis* in Brazil found 20% higher phenolics in high light and no effect of light on saponins (Sinimbu et al. 2012). We are not aware of other studies of saponin responses to light. The limited increase in saponins that we found may reflect some of the physiological constraints already noted, which lead to apparent canalization of young‐leaf development (Sinimbu et al. 2012). This differs from comparisons of mature leaves in understory versus canopy that found very large increases, on the order of threefold, for phenolics and tannins (Dominy et al. 2003).

In contrast, we found that tyrosine increased by 148% in gaps. N‐fixation is reported to be facultative in *Inga* such that N‐fixation is greater in gaps (Barron et al. 2011). Thus, in gaps, *Inga* may not be N‐limited and could afford to invest in a nitrogen‐based defense such as tyrosine, especially as tyrosine is catabolized at the end of leaf expansion (Lokvam et al. 2006).

### The relationship of leaf expansion rate to induced defenses

Although induction was limited, we found an interaction with the rate of leaf expansion such that only the two fast‐expanding species, *I. marginata* and *I. umbellifera*, showed an increase in phenolics in the presence of herbivores (Table 1, Herbivore presence × expansion rate, *P* < 0.05). Consistent with theory (Zangerl and Rutledge 1996; Ito and Sakai 2009), some empirical studies report that induction correlates with a low probability of attack (Zangerl and Rutledge 1996; Henery et al. 2008; but see Thaler and Karban 1997; Rasmann and Agrawal 2011). Our study supports the prediction that defenses are primarily constitutive when the probability of herbivore attack is high, as in tropical forests. However, the rate of leaf expansion, a trait unrelated to whole‐plant growth rate, varies among species and may be important for understanding induced responses. A shorter expansion phase may significantly reduce apparency or the probability of discovery by herbivores. For example, fast‐expanding *Inga* species spend <2 weeks in the vulnerable, young‐leaf stage while the leaves of slow‐expanding species take 2–4 weeks to grow from buds to their full size (Kursar and Coley 2003; Coley et al. 2005; Brenes‐Arguedas et al. 2006). In our study, 29% of fast‐expanding young leaves were discovered by herbivores versus 38% for slow‐expanding (Bixenmann et al. 2013) and extracts of the leaves of fast‐expanding species were, on average, less toxic in bioassays (Kursar and Coley 2003). These observations are consistent with the hypothesis that fast expansion correlates with lower probability of herbivore attack, less constitutive defense, and more induced defenses.

### Plasticity of defenses in *Inga*

Previously, we reported that chemical defenses among *Inga* species are divergent even for closely related species (Kursar et al. 2009; Endara et al. 2015). However, because plants manifest strong responses to environmental variation, it is important to distinguish whether divergent traits result from plastic responses such as induction by herbivory and light or from species‐level differences that are due to fixed (constitutive) traits (Ballhorn et al. 2011). The four *Inga* species in the present study differed in the mass of the phenolic, saponin, and tyrosine fractions (Fig. 2). Using a more rigorous metabolomics approach than in our previous study, we found that all four species also differed significantly in the composition of unique phenolic compounds (Figs. 3, 9A and S10). Moreover, given that both light and damage often induce very strong responses in plants, the absence of qualitative changes within species in phenolic composition in response to these factors (Figs. 3, S5, S9 and S10) supports our conclusion that interspecific differences are due to constitutive rather than plastic responses, and that within *Inga*, chemical defenses have undergone rapid evolution. On the other hand, our results also show that the population‐ or species‐level differences in certain components, such as tyrosine, could be due to plasticity.

## Conclusion

Although it is widely accepted that most species can and do induce defenses, we argue that the costs and benefits of phenotypic plasticity or induced defenses will differ in low‐ versus high‐risk environments. For a high risk of herbivory or low reliability of information about future herbivory, constitutive antiherbivore defenses may result in higher fitness and natural selection will result in a weak response to herbivory. The expanding leaves of tropical species represent an opportunity to test these theories under conditions of high risk. Focusing on one genus of trees from the tropics, we demonstrated that, while they can induce defenses, the increase is small. Thus, our results support a relatively untested component of the induced resistance hypothesis: high and consistent herbivore pressure should select for constitutive defenses.

## Conflict of Interest

None declared.

## Supporting information


**Figure S1**. Crude plant extract (A) and flavanoid extracts (B and C) from *Inga multijuga* reduced generalist herbivore growth relative to control (GRC).
**Figure S2.** Crude plant extract (A) and flavanoid extracts (B and C) from *Inga pezizifera* reduced generalist herbivore growth relative to control (GRC).
**Figure S3.** Crude plant extract (A) and flavanoid extracts (C and D) from *Inga marginata* reduced generalist herbivore growth relative to control (GRC).
**Figure S4.** Flavanoid extracts (C and D) and tyrosine (B) from *Inga umbellifera* reduced generalist herbivore growth relative to control (GRC).
**Figure S5.** Features that responded to herbivory that were selected by volcano plot analysis.
**Figure S6.** There was no significant effect of ant visitation on the three chemical classes: phenolics (A), saponins (B), and tyrosine (C).
**Figure S7.** The concentration (dry weight compound/dry weight of leaf tissue) of saponins (B) and tyrosine (C) increased when trees were found in gaps.
**Figure S8.** The concentrations of all chemical defenses (dry weight compound/dry weight of leaf tissue) decreased as the leaves matured.
**Figure S9.** Metabolite profiles obtained by UPLC‐ToF MS (positive mode).
**Figure S10.** Score plots of PCA on the influence of light and herbivory on the metabolic profile of different *Inga* species.
**Table S1**. Identity of significant features from the volcano plot analysis (Fig. S5).
