# Supplementary material for: High herbivore pressure favors constitutive over induced defense
Source: Ecol Evol. 2016 Jul 29;6(17):6037–49. doi: 10.1002/ece3.2208 (PMC5016630; doi:10.1002/ece3.2208)
Supplement: Supplementary file 1 — Figure S1. Crude plant extract (A) and flavanoid extracts (B and C) from Inga multijuga reduced generalist herbivore growth relative to control (GRC). Figure S2. Crude plant extract (A) and flavanoid extracts (B and C) from Inga pezizifera reduced generalist herbivore growth relative to control (GRC). Figure S3. Crude plant extract (A) and flavanoid extracts (C and D) from Inga marginata reduced generalist herbivore growth relative to control (GRC). Figure S4. Flavanoid extracts (C and D) and tyrosine (B) from Inga umbellifera reduced generalist herbivore growth relative to control (GRC). Figure S5. Features that responded to herbivory that were selected by volcano plot analysis. Figure S6. There was no significant effect of ant visitation on the three chemical classes: phenolics (A), saponins (B), and tyrosine (C). Figure S7. The concentration (dry weight compound/dry weight of leaf tissue) of saponins (B) and tyrosine (C) increased when trees were found in gaps. Figure S8. The concentrations of all chemical defenses (dry weight compound/dry weight of leaf tissue) decreased as the leaves matured. Figure S9. Metabolite profiles obtained by UPLC‐ToF MS (positive mode). Figure S10. Score plots of PCA on the influence of light and herbivory on the metabolic profile of different Inga species. Table S1. Identity of significant features from the volcano plot analysis (Fig. S5). [file ECE3-6-6037-s001.docx]

**Supporting Information**


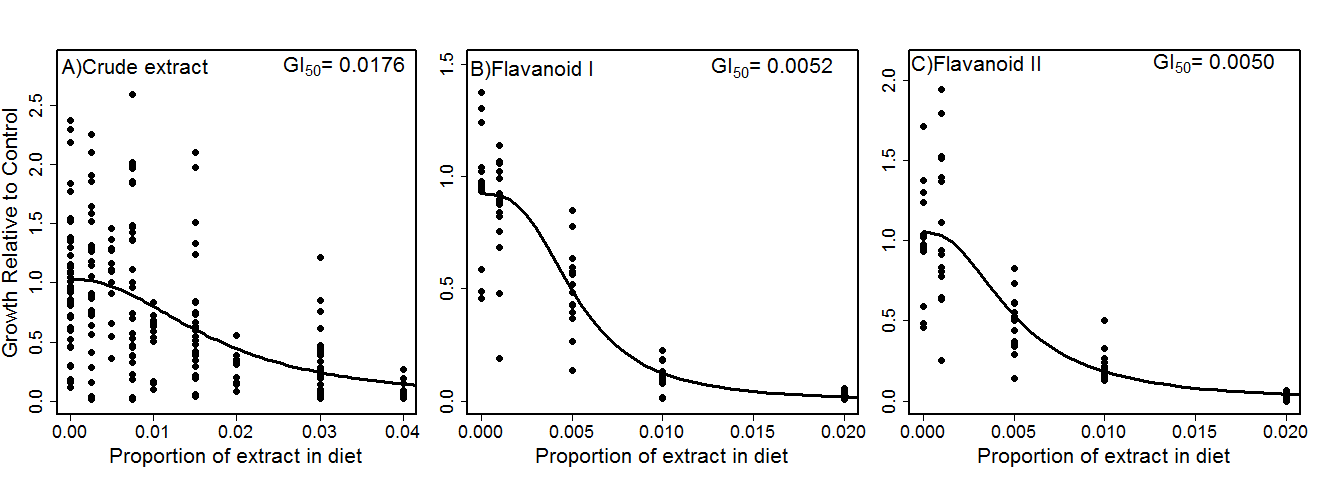


**Fig. S1**. Crude plant extract (A) and flavanoid extracts (B and C) from *Inga multijuga* reduced generalist herbivore growth relative to control (GRC). Growth inhibition 50 (GI_50_) is the concentration at which weight on the treatment diet is half that of the individuals fed a control diet. Thus, a lower GI_50_ suggests a more toxic diet. As the proportion of plant extract in the herbivore diet increased (x-axis), herbivore GRC decreases (y-axis). GI_50_s are reported when there is a significant relationship between extract concentration and GRC. Diet preparation, assay methods, data analysis, and GI_50_s of other *Inga* species are reported in Coley et al. (2005) and Brenes-Arguedas et al. (2006).


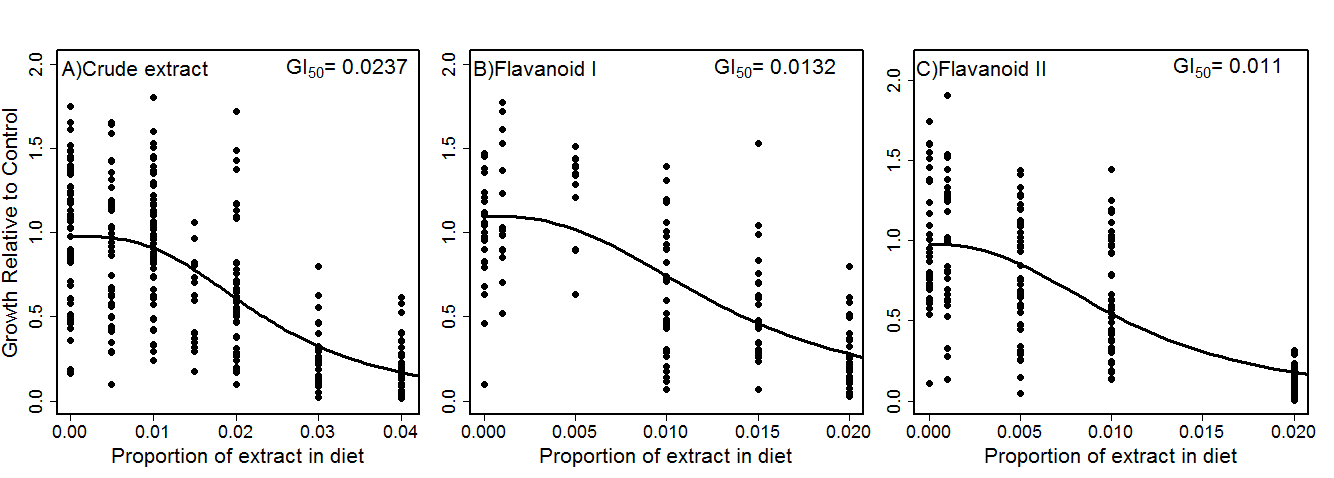


**Fig. S2.** Crude plant extract (A) and flavanoid extracts (B and C) from *Inga pezizifera* reduced generalist herbivore growth relative to control (GRC). Growth inhibition 50 (GI_50_) is the concentration at which weight on the treatment diet is half that of the individuals fed a control diet. Thus, a lower GI_50_ suggests a more toxic diet. As the proportion of plant extract in the herbivore diet increased (x-axis), herbivore GRC decreases (y-axis). GI_50_s are reported when there is a significant relationship between extract concentration and GRC. Diet preparation, assay methods, data analysis, and GI_50_s of other *Inga* species are reported in Coley et al. (2005) and Brenes-Arguedas et al. (2006).

**Fig. S3.** Crude plant extract (A) and flavanoid extracts (C and D) from *Inga marginata* reduced generalist herbivore growth relative to control (GRC). Saponins (B) from *I. marginata* did not affect herbivore growth. Growth inhibition 50 (GI_50_) is the concentration at which weight on the treatment diet is half of the individuals fed a control diet. Thus, a lower GI_50_ suggests a more toxic diet. As the proportion of plant extract in the herbivore diet increased (x-axis), herbivore GRC decreases (y-axis). GI_50_s are reported when there is a significant relationship between extract concentration and GRC. Diet preparation, assay methods, data analysis, and GI_50_s of other *Inga* species are reported in Coley et al. (2005) and Brenes-Arguedas et al. (2006).

**Fig. S4.** Flavanoid extracts (C and D) and tyrosine (B) from *Inga umbellifera* reduced generalist herbivore growth relative to control (GRC). Crude plant extract (A) from *I. umbellifera* did not significantly affect herbivore growth. Growth inhibition 50 (GI_50_) is the concentration at which weight on the treatment diet is half of the individuals fed a control diet. Thus, a lower GI_50_ suggests a more toxic diet. As the proportion of plant extract in the herbivore diet increased (x-axis), herbivore GRC decreases (y-axis). GI_50_s are reported when there is a significant relationship between extract concentration and GRC. Diet preparation, assay methods, data analysis, and GI_50_s of other *Inga* species are reported in Coley et al. (2005) and Brenes-Arguedas et al. (2006).


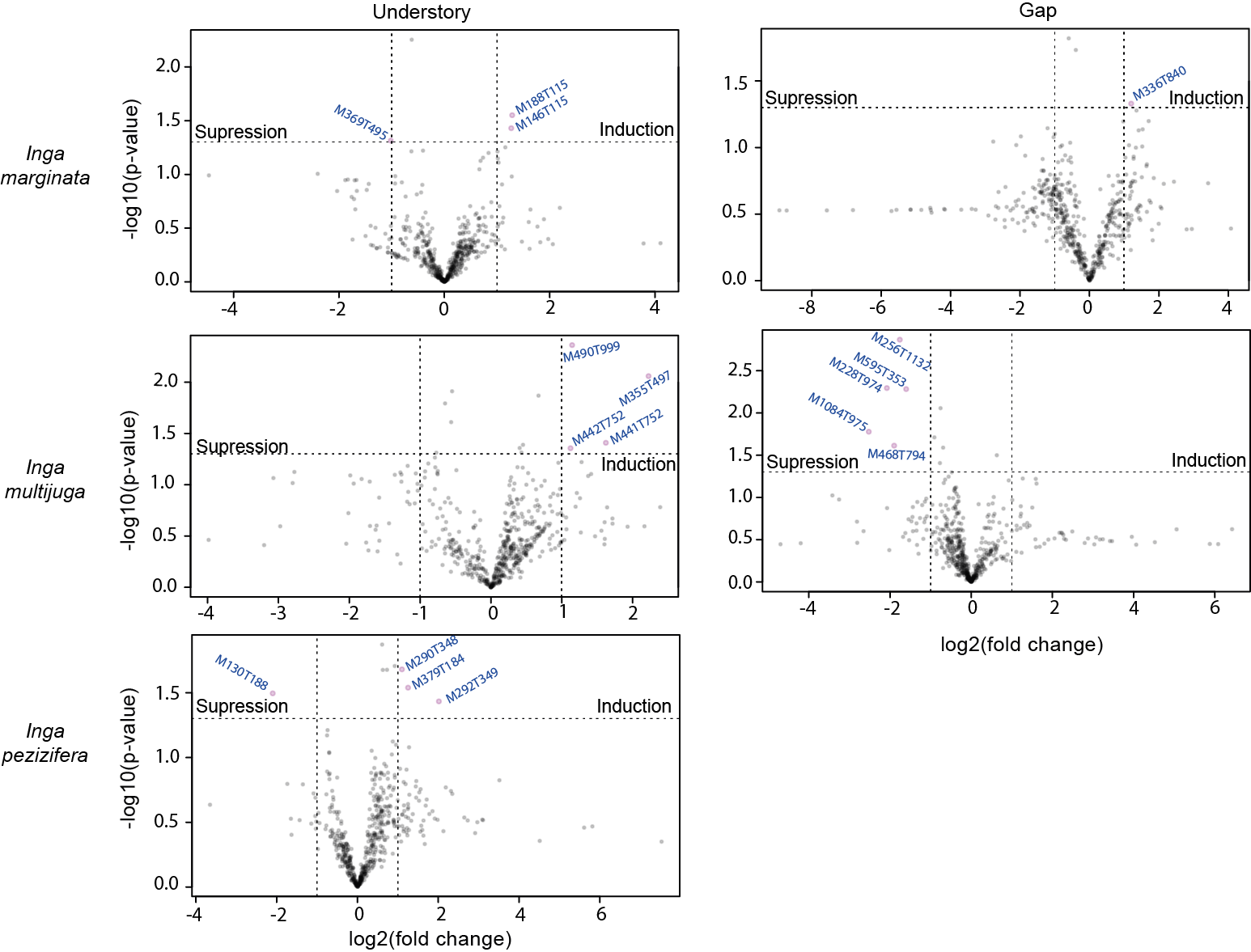
**Fig. S5**: Features that responded to herbivory that were selected by volcano plot analysis. The fold-change is plotted on the x-axis using a log 2 scale with a threshold of 2-fold change (dashed lines). The statistical significance of the change in each metabolite is indicated on the y-axis (log transformed p-values) with a threshold of p = 0.05. The red circles represent features that exceed these thresholds. The further from (0,0), the more significant the feature is. The significant features were evaluated by inspection of the UPLC-MS runs and the results are summarized in Table S1.

**Fig. S6.** There was no significant effect of ant visitation on the three chemical classes: phenolics (A), saponins (B), and tyrosine (C). White bars represent leaves with normal ant visitation and black bars represent leaves from which ant visitation was prevented.

**Fig. S7.** The concentration (dry weight compound / dry weight of leaf tissue) of saponins (B) and tyrosine (C) increased when trees were found in gaps. White bars represent plants located in gaps and black bars represent plants located in the understory. There was no effect of canopy on phenolics (A). P-values represent main effects of the forest canopy on the three chemical classes.

**Fig. S8.** The concentrations of all chemical defenses (dry weight compound / dry weight of leaf tissue) decreased as the leaves matured. White bars represent leaves that were 50-74% of the adult leaf sizes (size class 3). Black bars represent leaves that were 75-100% of adult leaf size but had not yet fully lignified into an adult leaf. P-values represent main effects of leaf size on the three chemical classes: phenolics (A), saponins (B), and tyrosine (C).

**
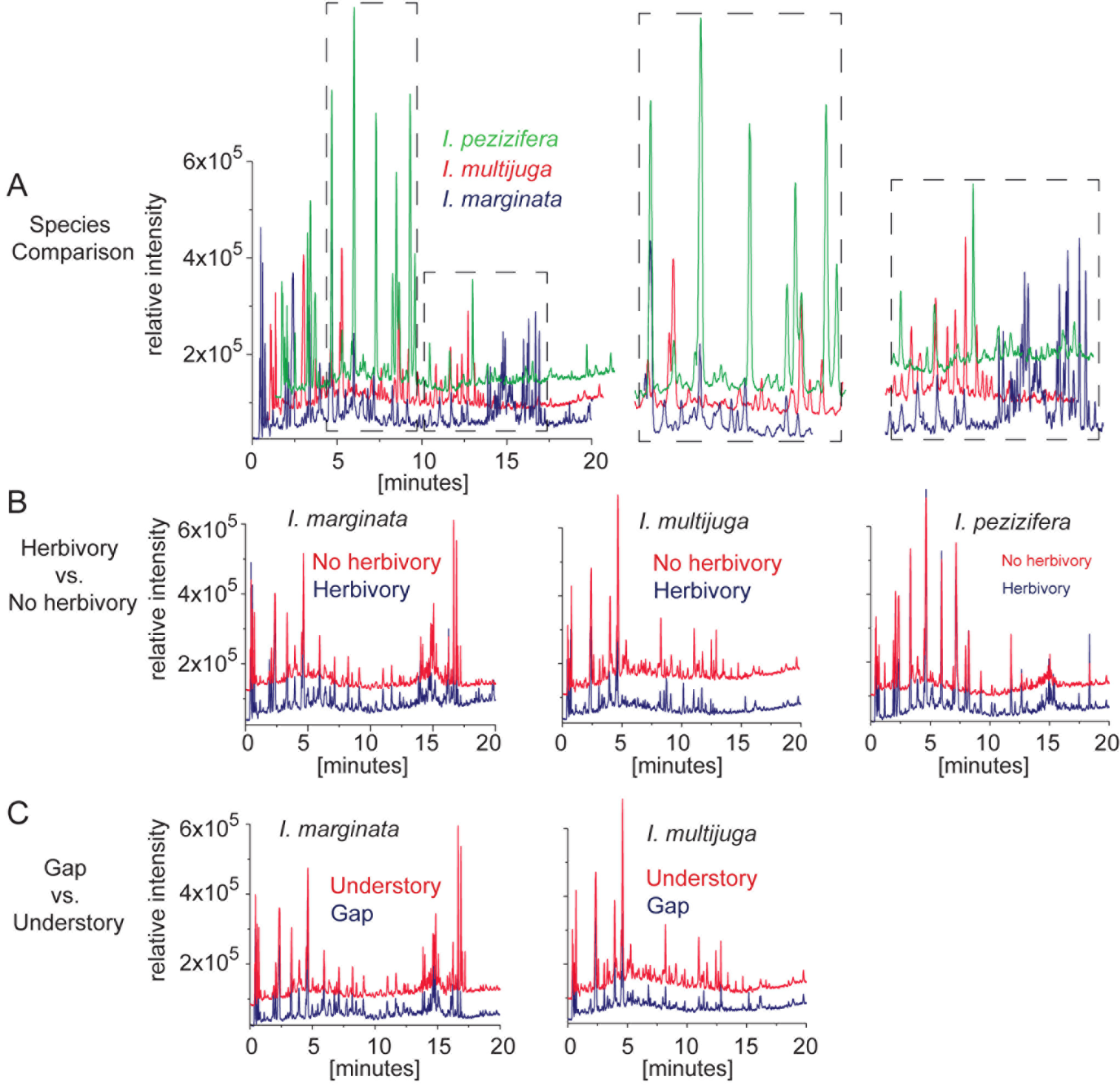
**

**Fig. S9.** Metabolite profiles obtained by UPLC-ToF MS (positive mode). (A) Left panel: Comparison of profiles from *Inga marginata, I. multijuga and I. pezizifera* in understory environments with herbivory. Profiles vary significantly among species. The two dashed boxes highlight regions that differ between species. These are expanded in the two right panels. (B) Effect of herbivory (red line represents plants to which herbivores were added, blue line *represents plants from which herbivores were removed) on the different *Inga* species in understory environment. No effect of herbivory is visible. No differences due to herbivory were observed in gaps (data not shown). (C) Effect of environment (red line represents plants found in understories, blue line represents plants found in gaps) on *Inga* species in absences of herbivory. No effect of environment was visible for plants exposed to herbivory (data not shown).
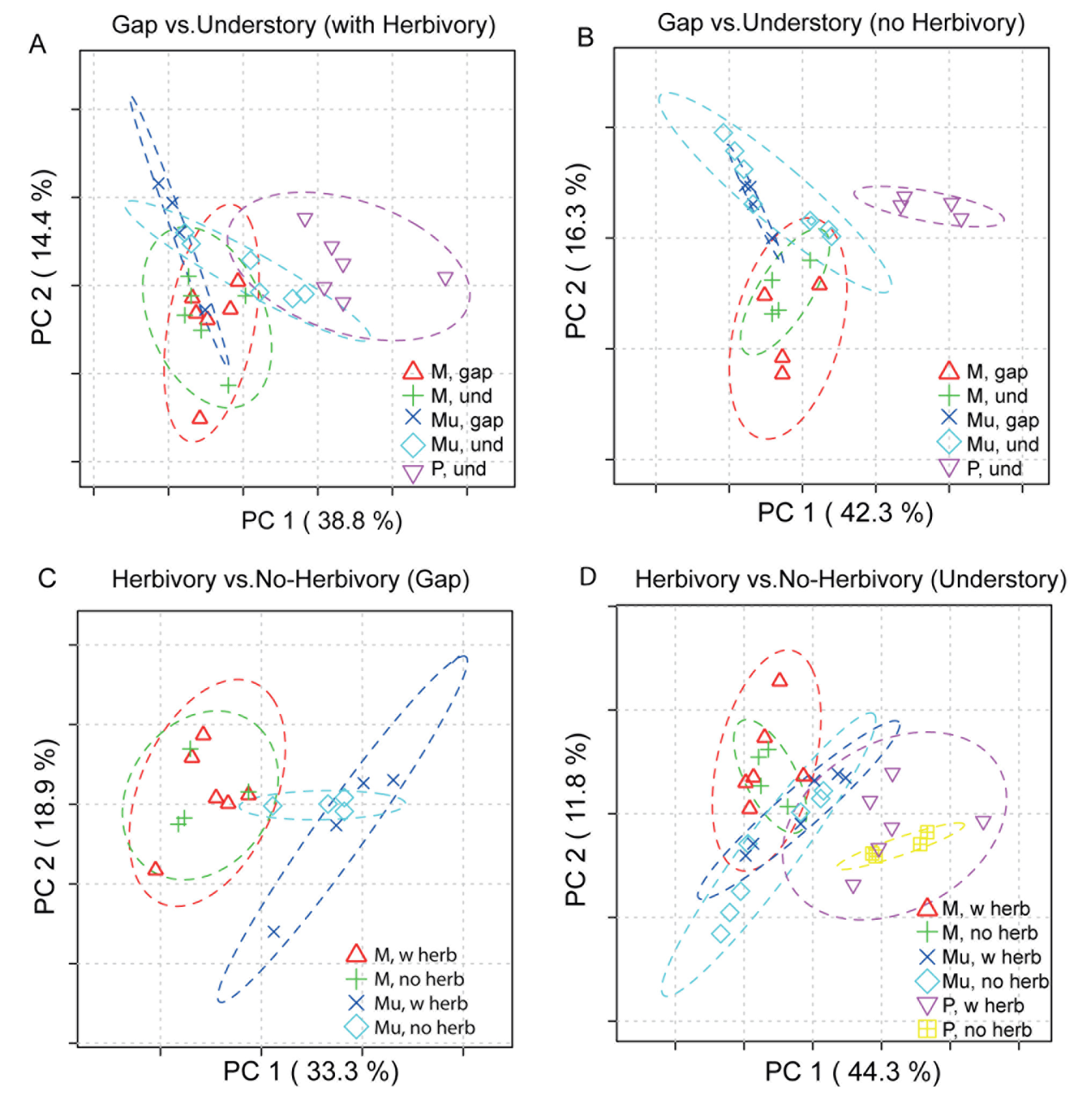


**Fig. S10.** Score plots of PCA on the influence of light and herbivory on the metabolic profile of different *Inga* species. (A) Comparison of *Inga marginata (M), I. multijuga (Mu), I. pezizifera (P)*, all with herbivory (+) in gap (G) and understory (U) locations. (B) Comparison of *Inga marginata (M), I. multijuga (Mu), I. pezizifera (P)* in a non-herbivory environment (-) in gap (G) and understory (U) locations (C) Comparison of *Inga marginata (M) and I. multijuga (Mu)* in an herbivory environment (+) and non-herbivory (-) environment in a gap (G). (D) Comparison of *Inga marginata (M) I. multijuga (Mu), I. pezizifera (P*) in an herbivory environment (+) and non-herbivory (-) environment in the understory (U). Dashed ovals represent the 95% confidence interval. Metabolite profiles are not different among treatments, however, there are distinguishable differences due to plant species.

**Table S1**. Identity of significant features from the volcano plot analysis (Figure S5). Most of the compounds are false positives from the data processing. False positives are low abundance features that were picked by the processing algorithm but are due to background noise.

|  | | Feature | log2(Fold Change) | -log10(p-value) | Fold Change | p-value | Comment |
| --- | --- | --- | --- | --- | --- | --- | --- |
| Understory | *Inga marginata* | M188T115 | -1.29 | 1.55 | 2.44 | 0.028 | N-containing compound |
|  |  | M146T115 | -1.27 | 1.43 | 2.41 | 0.037 | Fragment of M188T115 |
|  |  | M369T495 | 1.02 | 1.31 | 2.02 | 0.048 | False positive |
|  | *Inga multijuga* | M490T999 | -1.15 | 2.36 | 2.21 | 0.004 | False positive |
|  |  | M355T497 | -2.22 | 2.06 | 4.67 | 0.009 | False positive |
|  |  | M441T752 | -1.62 | 1.41 | 3.08 | 0.039 | False positive |
|  |  | M442T752 | -1.12 | 1.36 | 2.18 | 0.044 | False positive |
|  | *Inga pezizifera* | M290T348 | -1.10 | 1.68 | 2.15 | 0.021 | False positive |
|  |  | M379T184 | -1.25 | 1.54 | 2.38 | 0.029 | False positive |
|  |  | M130T188 | 2.10 | 1.50 | 4.28 | 0.032 | Unknown |
|  |  | M292T349 | -2.02 | 1.43 | 4.04 | 0.037 | False positive |
| Gap | *Inga marginata* | M336T840 | -1.20 | 1.33 | 2.30 | 0.047 | Unknown |
|  | *Inga multijuga* | M256T1132 | 1.76 | 2.87 | 3.38 | 0.001 | False positive |
|  |  | M228T974 | 2.08 | 2.29 | 4.22 | 0.005 | False positive |
|  |  | M595T353 | 1.60 | 2.28 | 3.04 | 0.005 | Unknown |
|  |  | M1084T975 | 2.52 | 1.78 | 5.75 | 0.017 | False positive |
|  |  | M468T794 | 1.90 | 1.61 | 3.73 | 0.024 | False positive |
